# Enhanced Green Strength in a Polycarbonate Polyol-Based Reactive Polyurethane Hot-Melt Adhesive

**DOI:** 10.3390/polym16233356

**Published:** 2024-11-29

**Authors:** Alejandra Moyano-Vallejo, María Pilar Carbonell-Blasco, Carlota Hernández-Fernández, Francisca Arán-Aís, María Dolores Romero-Sánchez, Elena Orgilés-Calpena

**Affiliations:** INESCOP Footwear Technology Centre, Alemania 102, 03600 Elda, Alicante, Spain; pcarbonell@inescop.es (M.P.C.-B.); chernandez@inescop.es (C.H.-F.); aran@inescop.es (F.A.-A.); mdromero@inescop.es (M.D.R.-S.); eorgiles@inescop.es (E.O.-C.)

**Keywords:** green strength, reactive polyurethane hot-melt adhesive, bio-based polycarbonate polyol, sustainable adhesive, thermoplastic polyurethane

## Abstract

This study aimed to enhance the initial adhesion performance of reactive polyurethane hot-melt adhesives by using a bio-based polycarbonate polyol instead of traditional polyester or polyether polyols and by incorporating thermoplastic polyurethane (TPU) in varied proportions. Adhesives synthesized from bio-based polycarbonate polyols and polypropylene glycol with MDI as the isocyanate were characterized chemically, thermally, and mechanically (FTIR, DSC, plate–plate rheology, DMA, and T-peel strength test). Adding 10–15 wt.% TPU significantly improved green strength and initial adhesion at room temperature and after accelerated cooling. The bio-based polycarbonate polyol promotes superior flexibility at low temperatures compared to fossil-derived alternatives, aligning with sustainability objectives. The results showed that 10 wt.% TPU maximized green strength without compromising flexibility, whereas 15 wt.% TPU, though enhancing adhesion, reduced flexibility due to increased crystallinity. T-peel tests on footwear materials indicated that all the adhesives exceeded the EN 15307:2015 requirements, with the highest peel strength achieved after curing. These findings highlight the benefit of bio-based polycarbonate polyols and TPUs in achieving strong, flexible, and eco-friendly adhesives suitable for demanding applications.

## 1. Introduction

Reactive polyurethane hot-melt adhesives (HMPURs) represent an advanced solution in adhesive technology. These adhesives combine the advantages of traditional hot-melt adhesives with the enhanced bonding strength of reactive chemistry. These adhesives are initially applied as thermoplastics in their molten state and cool to form a bond but then undergo a secondary curing process via a reaction with moisture in the air or substrates, transforming into a cross-linked, thermoset polymer [1]. This dual bonding mechanism provides superior strength, flexibility, and resistance to heat, moisture, and chemicals, making HMPURs ideal for demanding applications in industries such as automotives, electronics, footwear, and furniture manufacturing [2].

When selecting adhesives for industrial applications, factors such as bonding efficiency, durability, and environmental impact are critical considerations. Compared to water-based, solvent-based, and conventional hot-melt polyurethane adhesives, HMPURs offer several advantages. Unlike water-based adhesives, HMPURs do not require water evaporation for curing, enabling faster bonding processes and higher efficiency in production lines. Additionally, they avoid the environmental concerns and flammability risks associated with solvent-based adhesives due to the use of organic solvents. While traditional hot-melt adhesives provide rapid bonding, they lack the durability and temperature resistance of HMPURs. The precise control of the curing process is key, especially when bonding large surfaces, as uneven moisture exposure can affect the final bond strength. Crystallization in HMPURs also hinders moisture penetration into the system, thereby compromising adhesive performance [3]. Additionally, they tend to be more expensive than water-based or solvent-based adhesives. Another limitation is that HMPURs do not offer the same high green strength as solvent- or water-based PU adhesives, which often achieve this by adding a cross-linker right before application, which rapidly increases the molecular weight of the chains, resulting in good initial adhesion.

Building on the challenges of achieving high green strength in HMPURs, it is essential to consider the factors influencing this property and their implications for adhesive performance. Generally, the green strength of a polyurethane adhesive is related to the increase in the molecular weight and crystallization of the soft segments [1,4]. Another factor that can cause an effect is viscosity because the interface adhesion strength between the adhesive and the substrate will increase as the viscosity of the adhesive increases. The latter is of particular importance in HMPURs because they are designed to have a low melt viscosity for facile handling and applications and, thus, an implied generally lower molecular weight, another factor facilitating poor green strength after cooling [5]. Viscosity ranges for HMPURs vary depending on the application method and temperature. Between 120 and 190 °C, which are standard working temperatures, viscosities between 5000 and 10,000 mPa·s are suitable for spray application while viscosities above 10,000 mPa·s (and up to 100,000 mPa·s with special equipments) can be used for roller or curtain coaters.

Among the many efforts to address the challenges in green strength, the incorporation of rosin, a natural resin derived from pine trees, has proven to be an effective approach in polyurethane hot-melt formulations. Rosin and its derivatives are well known for their intrinsic adhesive qualities and their capacity to increase the viscosity of the polymer matrix, which facilitate greater surface contact during application [6,7]. Introducing rosin into polyurethane formulations has shown the potential to improve the initial adhesion on various substrates, including plastics and composite materials, enabling the rapid and efficient handling of assembled parts in high-performance production environments [8]. Optimizing polyurethane systems with rosin can also enhance other adhesive properties such as flexibility and moisture resistance. These characteristics can be tailored by adjusting the type and proportion of rosin in the blend, enabling the development of application-specific products in industries such as automotives, packaging, and construction. Xie et al. [1] studied the effect of adding a rosin-based chain extender to HMPURs to increase their green strength. They found that green strength first increases and subsequently decreases with an increase in the chain extender content. This is attributed to the formation of substantial amounts of low-molecular-weight polyurethane prepolymers, which weakens the crystallization ability of HMPURs and leads to lower green strength.

Another approach is based on incorporating substances with hydroxyl terminations to the formulations, such as monomers, copolymers, etc. In this sense, the use of acrylate copolymers to increase green strength has also been described in the literature. Sun et al. [5], for example, synthetized a pentaerythritol diacrylate that was added in a proportion between 0 and 10 wt.% to an HMPUR as a reactive component after prepolymer synthesis. They found that the viscosity, elastic modulus, and tensile strength increased with the incorporation of the diacrylate, until a maximum of 7 wt.%, because the set time for the proper application of the adhesive was too short. High-molecular-weight acrylic polymers were used by Slark [9] to promote chain entanglements and the enhancement of green strength while retaining similar melt viscosity. However, when an acrylic polymer has more than two hydroxyl groups, it is easy to cause cross-linking and gelation, which will result in control difficulty during the polycondensation process [10,11].

It is also possible to achieve high initial adhesion using crystalline materials such as polyester diols, although this substantially limits the achievable open time [9]. In this sense, Bhagavathi et al. [12] studied the effect of incorporating microcrystalline cellulose or sawdust powder into single-component moisture-curable polyurethane adhesives. When added to blends of polyols and isocyanates during the synthesis of polyurethanes, given the high reactivity of these fillers with isocyanate, higher curing speeds are obtained, resulting in high initial adhesion. On the other hand, the fillers also acted as reinforcement, resulting in higher shear strength compared to the unfilled adhesive, but improvements strongly depend on the dispersion method and the added load content.

Expanding on the role of polyols in HMPUR formulations, the selection of specific polyol types significantly influences the performance and application suitability of these adhesives. HMPURs are typically formulated with blends of polyester, polyether, and, less commonly, polycarbonate polyols (PCCs). Polyester polyols provide excellent adhesive properties, high tensile strength, and temperature resistance, imparting a crystalline character to the adhesive. However, they have limited hydrolytic stability. Conversely, a significant portion of HMPURs are based on polyether polyols, which are valued for their low cost, ease of handling, and good hydrolytic, as well as chemical, resistance. These polyols enhance the flexibility of an adhesive, though they are prone to oxidation when exposed to UV light [13].

In this context, PPC polyols have emerged as an attractive option due to their exceptional resistance to hydrolysis, thermal ageing, and environmental degradation. These polyols improve the durability of HMPURs by providing high stability in humid conditions and at extreme temperatures, enhancing the mechanical properties of an adhesive through strong bonds that increase cohesion without sacrificing flexibility. Although their synthesis is more complex and costly compared to that of polyester or polyether polyols, PCCs are gaining importance in applications requiring superior performance and higher resistance to degrading factors such as continuous exposure to moisture and UV light [14,15].

Carbonell et al. [16] developed polyurethane adhesives using PCCs derived from CO_2_, exploring their potential as sustainable alternatives in the footwear, wood, and furniture sectors. This study examines the mechanical, adhesive, and stability properties of these adhesives compared to those of traditional formulations, highlighting how incorporating CO_2_-based polycarbonate polyols not only reduces reliance on fossil-based materials but also enhances durability for demanding applications. The research underscores the feasibility of these adhesives for industrial applications and their contributions to sustainability in these industries.

Liu et al. [3] studied a series of HMPURs based on PCCs derived from CO_2_ with different carbonate linkage contents as an alternative to HMPURs from non-renewable petroleum-based polyols. The adhesives consisted of polyol mixtures of 50 wt.% of polyester polyols and 50 wt.% of PPC polyols with 99% carbonate linkages, 65% carbonate linkages, or a 1:1 mixture of both. As an isocyanate, MDI was used. They found that carbonate linkage content has no effect on the moisture curing rate or thermal stability, and that increasing the carbonate content of PPC leads to a significant increase in adhesion strength, mechanical strength, and modulus. They also demonstrated that carbonate groups form hydrogen bonds with urethane groups hindering the crystallization of the adhesive; therefore, higher carbonate linkage content also leads to lower initial strength (after 5 min of curing) in lap shear tests of iron, steel, and aluminum.

Fuensanta et al. studied polyurethane formulations based on mixtures of polyester and polycarbonate diols to achieve self-healing properties, with a focus on polyol compatibility and segmented structures [17,18]. Research highlights that incorporating polycarbonate diols enhances the thermal stability, elasticity, and hydrolytic resistance of these polyurethanes, making them highly suited for applications like protective coatings and adhesives.

The present study investigates the development of polycarbonate-based reactive polyurethane hot-melt adhesives incorporating different proportions of a thermoplastic polyurethane (TPU). The objective is to examine how polycarbonate diol and TPU contents influence the properties of the adhesives, with a particular focus on strategies to improve initial adhesion. The incorporation of the TPU is intended to reduce the setting time and open time, providing green strength that is crucial in fast production processes or when large surfaces are to be joined together. The use of polycarbonate diols seeks to improve the mechanical performance of adhesives in extreme conditions with respect to that provided by traditional hot-melt adhesives. This can be useful in construction (sealing of facades and joints), appliances (bonding of refrigeration components), aerospace (fuselage assembly), renewable energy (mounting of wind turbines and solar panels), and textiles (footwear and technical clothing for cold climates), where maintaining adhesion in extreme cold conditions is required. Additionally, bio-sourced polycarbonate polyols offer a sustainable alternative, aligning with eco-friendly innovations by reducing dependence on fossil-based polyols commonly used in conventional adhesive formulations and offering enhanced flexibility at low temperatures.

## 2. Materials and Methods

### 2.1. Materials

Reactive polyurethane hot-melt adhesives were synthetized by using a blend of a polyether of fossil origin and a bio-based polycarbonate polyol. For the polyol of fossil origin, polypropylene glycol (PPG 425, Quimidroga S.A., Barcelona, Spain) (*Mw* = 425 g/mol, I_OH_ = 250–270 mg KOH/g) was used. Eternacoll BIO UD-140DF (*Mw* = 1400 g/mol, I_OH_ = 80 mg KOH/g, 83% biobased content according to USDA-Certified Biobased Product) was kindly provided by UBE Corporation Europe as a supplier (Castellon, Spain) and was used as the bio-based polycarbonate diol. For the diisocyanate, 4,4′-diphenylmethane diisocyanate (Sigma Aldrich, Barcelona, Spain) was used. Pearlbond 521, a polycaprolactone copolyester-based polyurethane, was used as the TPU (Lubrizol Advanced Materials Spain S.L., Barcelona, Spain) (Shore hardness = 54 D, Tm by DSC = 58 °C, melt viscosity at 130 °C = 400–900 Pa·s).

### 2.2. Methods

#### 2.2.1. HMPUR Synthesis

**Figure 1:**
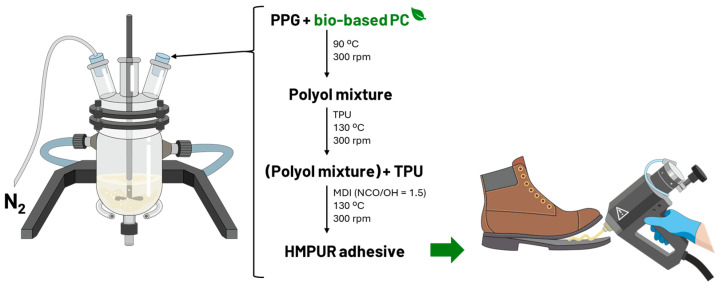
HMPUR synthesis scheme.

Before their use, polyols were previously vacuum-dried at 80 °C for 4 h. Adhesives were prepared via the prepolymer method [19,20] with a NCO/OH ratio of 1.5 based on stoichiometric calculations. In the first stage, polyols were melted and mixed under continuous mechanical stirring (300 rpm) at 90 °C in an inert nitrogen atmosphere. Then, the isocyanate was added and mixed for reaction until reaching the desired free isocyanate content, which was determined by the dibutyl amine method [21]. At this point, the reaction was stopped, and the adhesive was stored in a hermetically sealed cartridge. In the formulations with the TPU, it was added to the polyol mixture by raising the temperature to 130 °C and maintaining this temperature for the remainder of the synthesis (Figure 1). The characterization of the uncured adhesive was carried out 24 h after synthesis. To carry this out, the adhesive was heated to 140 °C using a heat gun, and a sample was deposited on a Teflon plate until a film of non-uniform thickness was formed. Fully cured samples were prepared by allowing the adhesive to cure at 23 °C and 50%RH for 7 days.

The reference reactive polyurethane hot-melt adhesive (PUR) was obtained via equal mixing of the bio-based polycarbonate polyol and polypropylene glycol. Different amounts of the TPU were then added to prepare adhesives PUR-10 (10 wt.% of the TPU) and PUR-15 (15 wt.% of the TPU), as shown in Table 1.

#### 2.2.2. Adhesive Characterization

*Fourier-transform infrared spectroscopy (FTIR)*: The chemical properties of adhesives were analyzed using a Cary 630 infrared spectrometer (Agilent Technologies, Santa Clara, CA, USA). The analysis was conducted using attenuated total reflection (ATR) technology, with 16 scans performed at a resolution of 4 cm^−1^. The adhesive film was placed in direct contact with the prism for testing. Two specimens per sample were evaluated to ensure reproducibility of the results

*Thermogravimetric analysis (TGA)*: The thermal behaviour of adhesives after full curing, stored for 7 days at 23 °C and 50% relative humidity, was analyzed using a TGA 2 STARe System thermobalance (Mettler-Toledo AG, Schwerzenbach, Switzerland). Approximately 7–10 mg of each sample was placed on an aluminum crucible and subjected to a heating program from 30 to 600 °C at a rate of 10 °C/min under an inert nitrogen atmosphere (30 mL/min). Two specimens per sample were tested to confirm the reproducibility of the results.

*Plate–plate rheology*: To assess the processability of uncured adhesives, their viscoelastic properties were evaluated using a Kinexus Pro+ rheometer (Malvern Analytical, Malvern, UK) with a 25 mm diameter upper plate. The analysis involved a temperature ramp from 160 °C to 25 °C at a cooling rate of 5 °C/min, conducted at a constant frequency of 1 Hz and a controlled strain of 1%. The gap between the plates was maintained at 0.4 mm.

*Differential scanning calorimetry (DSC)*: To investigate the thermal transitions of fully cured adhesives, a DSC3+ Stare Systems calorimeter (Mettler-Toledo AG, Schwerzenbach, Switzerland) was used. Aluminum pans containing 9–12 mg of sample were analyzed using a heating rate of 10 °C/min. The analysis included an initial heating cycle from −15 to 100 °C (first heating), followed by a second heating cycle from −65 to 100 °C. All measurements were performed under a nitrogen atmosphere with a flow rate of 50 mL/min. Two specimens of each sample were tested to confirm reproducibility.

*Dynamic mechanical thermal analysis (DMTA)*: The viscoelastic behaviour of fully cured adhesives was assessed with DMA-Q800 equipment (TA Instruments, New Castle, DE, USA) in single-cantilever mode. Adhesive films measuring 35 mm × 13 mm × 3 mm were analyzed under a nitrogen atmosphere with a heating rate of 5 °C/min, covering a temperature range from −80 to 100 °C. The tests were performed at a constant frequency of 1 Hz, an amplitude of 20 μm, and a strain of 0.5%.

*T-peel strength test*: The adhesion properties were assessed following the EN 1392:2006 standard [22]. Footwear reference materials measuring 150 × 30 mm, including soling material (vulcanized SBR-2 rubber, Proyección Europlan XXI, Elda, Spain) and upper material (chrome-tanned split leather, Palomares Piel S.L., Elda, Spain), were joined for testing. Prior to adhesive application, both materials underwent surface preparation. The split leather was roughened at 2,800 rpm using a P100 aluminum oxide abrasive cloth (Due Emme Abrasivi, Pavia, Italy) and a roughing machine (Superlema S.A., Zaragoza, Spain). The SBR rubber was roughened and halogenated using a 2 wt.% trichloroisocyanuric acid solution in ethyl acetate (Insoco, Alicante, Spain). The HMPUR was applied at 140 °C on the rubber test specimen using a heat gun. After 30 min of adhesive application, simulating standard industrial footwear production, the substrates were activated with infrared radiation at 80 °C using a CAN 02/01 temperature-controlled heater (AC&N, Elda, Spain). The materials were then joined, and pressure of 1.8 bar was applied for 10 s, ensuring a consistent adhesive film thickness of 0.53 ± 0.01 mm. Adhesion performance was evaluated in two stages: initial adhesion was evaluated 5 min after joint formation and after 5 min of sudden cooling (10 min at −10 °C in a refrigerating chamber). Final adhesion was measured after storing the adhesive joints for 7 days at 23 °C and 50% relative humidity. T-peel strength tests were conducted using an Instron 34TM–10 universal testing machine (Instron Ltd., Buckinghamshire, UK) at a crosshead speed of 100 mm/min. Five specimens per adhesive were evaluated, and failure mode was assessed according to ISO 17708:2018 standard [23].

## 3. Results and Discussion

Reactive polyurethane hot-melt adhesives were synthesized by using a bio-polycarbonate diol and different amounts of a TPU—0 wt.% (PUR), 10 wt.% (PUR-10), and 15 wt.% (PUR-15)—to achieve initial bond strength. The composition included polypropylene glycol, which provides flexibility and elasticity, while the bio-based polycarbonate diol contributed to durability and thermal stability, supporting the sustainability of the adhesive. The incorporation of the TPU can help to increase crystallinity and shorten both the set time and open time of the adhesive, improving the initial bond strength [24]. Finally, 4,4′-MDI acts as a cross-linking agent, forming the polymeric matrix and influencing the overall mechanical properties and thermal resistance of the adhesive. The specific composition is detailed in Table 1.

The chemical structure of the HMPURs was assessed via FTIR analysis, and the assignment of bands was carried out by considering the previous literature [14,15,19,20,25,26,27]. All adhesives showed a small band at 3306 cm^−1^, corresponding to primary amine stretching; bands between 2917 and 2850 cm^−1^, from C-H stretching from aliphatic chains from the soft segments; and bands corresponding to the MDI at 1595, 1456, and 1410 cm^−1^, attributed to C=C in plane vibrations of the aromatic rings. A peak at 1531 cm^−1^ from N–H and C–N from urethane, a peak at 1735 cm^−1^ from C=O stretching due to the carbonyl group from the polycarbonate polyol and from the urethane, and a peak at 1232 cm^−1^ from OC(O)O stretching from carbonate groups were also observed.

**Figure 2:**
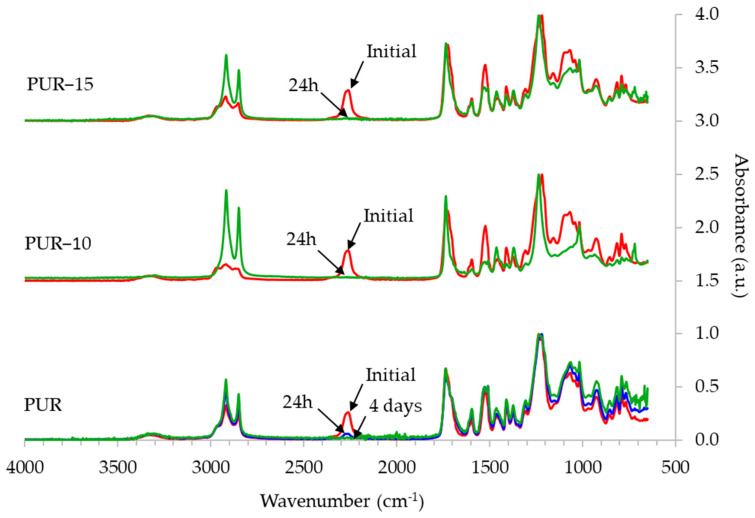
Comparison of the FTIR spectra of HMPURs just after synthesis (Initial) and after 24 h or 4 days of curing at 23 °C and 50% relative humidity.

Adhesive cross-linking was followed by monitoring the disappearance of the free isocyanate band at 2256 cm^−1^ via FTIR analysis (Figure 2). This approach is broadly used in the literature to study moisture-curing polyurethanes [3,4]. As observed in Figure 1, the peak at 2256 cm^−1^ decreased significantly for the PUR (without the TPU) after curing for 1 day but does not disappear completely until 4 days after curing started. In this sense, the curing of this adhesive is in line with the results previously described in the literature for HMPURs based on polycarbonate polyols [19,28,29]. On the other hand, for samples PUR-10 and PUR-15, the presence of the TPU in the formulation seems to accelerate the initial curing process, as the free isocyanate band is not observed after only 24 h of curing.

**Figure 3:**
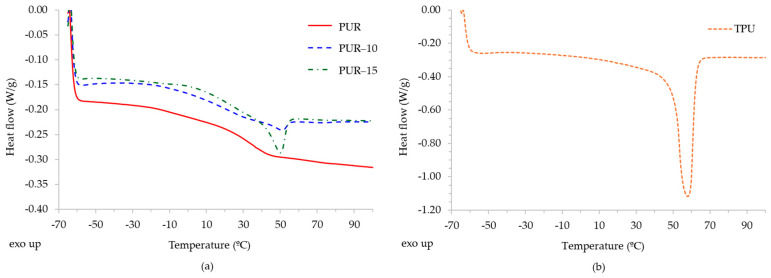
Comparison of DSC curves (second heating run) of the adhesives as a function of TPU content (**a**) and of the TPU (**b**).

The thermal behaviour of the HMPURs was evaluated by using differential scanning calorimetry (DSC) and thermogravimetric analysis (TGA). The DSC analysis was conducted to compare the thermal properties of the HMPURs (PUR-10 and PUR-15), which exhibit initial adhesion due to the TPU, with those of reference polyurethane adhesive without initial adhesion (PUR). Therefore, Figure 3 shows the DSC curves of the second heating run of the adhesives and of the TPU, with the data summarized in Table 2. The adhesive without the TPU (PUR) shows predominantly amorphous behaviour with a glass transition temperature (Tg) of −5.41 °C. The addition of the TPU (PUR-10 and PUR-15) displaces the Tg in the adhesive matrix, influenced by the Tg of the TPU itself (−18.76 °C), indicating partial miscibility with the polycarbonate diol and adding stiffness. A melting process appears around 50 °C, close to the melting temperature (Tm) of the TPU (57.99 °C), although with lower enthalpy (ΔHm), indicating partial crystallization within the matrix. This partial crystallization could improve the initial bond strength by accelerating crystallization during initial curing, which is critical for immediate bonding applications in the footwear industry and other sectors.

**Figure 4:**
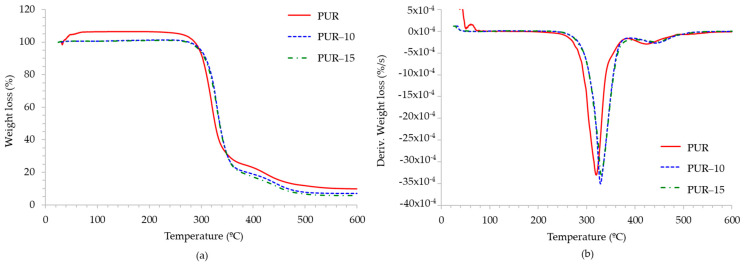
Comparison of the TGA (**a**) and DTGA (**b**) curves of the adhesives.

In relation to TGA, Figure 4 shows the TGA and TGA derivative (DTGA) curves of HMPURs with and without the TPU. Table 3 summarizes the decomposition temperatures, weight loss, and final residue after complete degradation under a nitrogen atmosphere. These adhesives display two main thermal degradation stages: the first decomposition stage (T1), around 319 °C, is mainly associated with the decomposition of polycarbonate units, while the second degradation stage (T2), which occurs at 425 °C, is related to the decomposition of soft segments from both polyols and from the TPU [17,19,28,29]. After TPU incorporation, both stages are shifted to higher temperatures (330–331 °C and 448–454 °C, respectively), indicating improved thermal stability. This could be related to the lower content in carbonate groups that decomposed earlier. The incorporation of the TPU also improves char formation, as evidenced by the lower residue content. This is consistent with the lower proportion of polyols in the mixture, which are the main contributors to the total hydrocarbon content.

**Figure 5:**
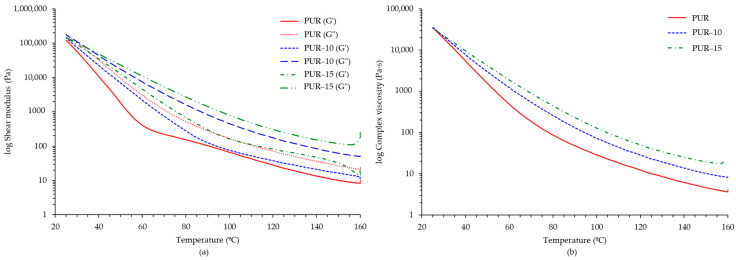
Results of plate–plate rheology experiments of adhesives. Comparison of the variation in the elastic (G′) and viscous moduli (G″) as a function of temperature (**a**) and comparison of the variation in the complex viscosity as a function of temperature (**b**).

To better understand the thermal and mechanical behaviour of the HMPURs, the rheological properties were evaluated as a function of the temperature of the adhesives after curing by using plate–plate rheometry. Figure 5 shows the evolution of the elastic modulus (G′) and viscous modulus (G″), as well as the complex viscosity of the adhesives, as a function of temperature in plate–plate rheology experiments. As shown in Figure 5a, the incorporation of the TPU into the adhesive formulation produces a clear rise in the G′ and G″ values, which is more noticeable for the higher TPU content, indicating an improvement in the mechanical and thermal properties of the adhesive. The PUR shows the lowest G′ values, reflecting lower stiffness and stability at higher temperatures. In contrast, the PUR-10 and PUR-15 adhesives show greater thermal stability, with PUR-15 showing the highest G′ values and a more gradual decrease in the elastic modulus with an increase in the temperature, indicating better thermal and structural resistance. In all cases, the elastic modulus is higher than the viscous modulus, indicating a predominantly elastic behaviour. The more stable viscoelastic performance observed in PUR-10 and PUR-15 compared to the PUR without the TPU highlights their suitability for applications requiring both mechanical strength and thermal stability [18]. The variation in complex viscosity as a function of the temperature of the adhesives is shown in Figure 5b. Melt viscosity increased with TPU concentration, but the values obtained are maintained in a suitable range for adhesive applications (i.e., heated guns). Between 120 and 160 °C, viscosity ranges are as follows: 3600–12,400 mPa·s for PUR; 8000–28,300 mPa·s for PUR-10; 17,600–50,400 mPa·s for PUR-15. In this sense, PUR and PUR-10 could be applied by spray or roller, while PUR-15 would not be suitable for spray applications due to its high viscosity.

**Figure 6:**
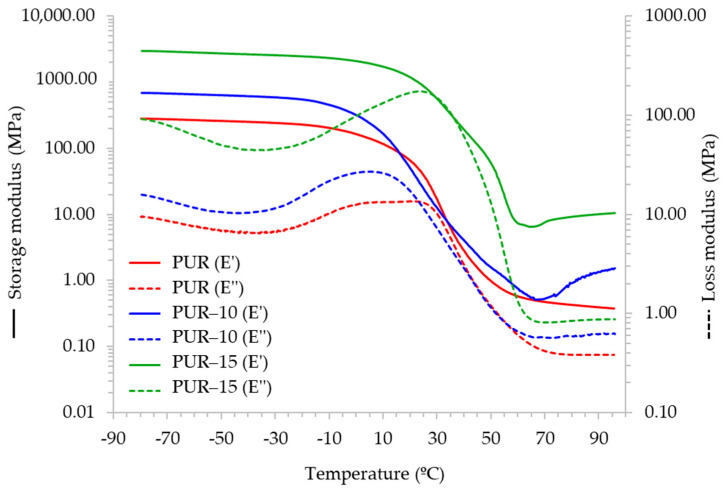
Variation in the storage (E′) and loss moduli (E″) of the adhesives with temperature.

**Figure 7:**
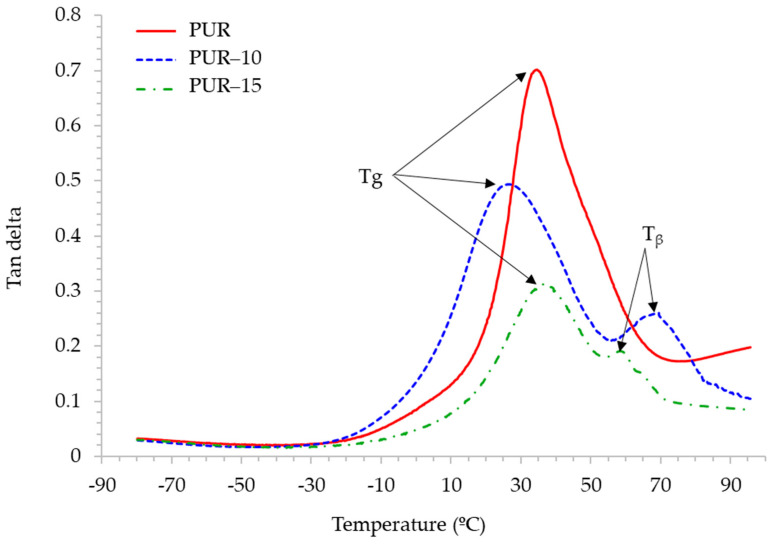
Variation in the tan delta of the adhesives with temperature.

Polycarbonate polyols are known to promote good flexibility at low temperatures in polyurethane structures [17,19,30]. To study the viscoelastic properties of adhesives at low temperatures, DMA studies were conducted. Figure 6 shows the evolution of the storage modulus (E′) and loss modulus (E″) for the adhesives as a function of temperature, and Figure 7 shows the variation in the tan delta. PUR shows good flexibility at a low temperature, with an elastic modulus around 252 MPa at −40 °C and a glass transition temperature (Tg) of 36 °C (Table 4). When a 10 wt.% of the TPU is added to the formulation, the flexibility at −40 °C decreases and phase separation occurs, as evidenced by the appearance of a secondary transition (T_β_) at a higher temperature (70 °C). The incorporation of a 15 wt.% of the TPU to the polycarbonate-based HMPUR promotes flexibility at a low temperature.

The ratio of the loss modulus to the storage modulus is defined as the damping factor or loss factor and is denoted as tan δ. Tan δ indicates the relative degree of energy dissipation or damping of the material. For example, a material with a tan δ > 1 will exhibit more damping than a material with a tan δ < 1 because the loss modulus is greater than the storage modulus in the former, which means that the energy dissipating viscous mechanisms will have a greater influence on the final properties of the material [31]. As is shown in Figure 6, there is a decrease in tan delta with the incorporation of the TPU, indicating a decrease in the damping properties with respect to the adhesive without the TPU. On the other hand, when crystalline and amorphous phases coexist in a material, then a reduced damping peak corresponds to an increased degree of crystallinity because these crystalline regions act as cross-linking points that inhibit the sliding motion of the molecular chains [32,33]. The adhesives under study present crystalline and amorphous phases, so it can be deduced that the incorporation of the TPU increases the crystallinity of the formulations.

In the footwear industry, the upper-to-sole joint represents one of the most critical connections due to the substantial stress that it endures, often under varying environmental conditions that may compromise its integrity. Consequently, strong adhesive bonds are essential to ensure durability and performance under these demanding conditions.

To simulate the performance of upper-to sole bonds in footwear, the T-peel strength test was performed on joints by using leather/HMPURs with and without TPU/SBR joints. This configuration provides a representative model for assessing adhesion under conditions relevant to footwear applications. The T-peel strength test results (Table 5 and Table 6) show the impact of TPU content on the initial and final adhesion of the HMPURs. In many industrial applications, several production steps involving mechanical stresses in the adhesive bond are interlinked; therefore, high initial adhesion or green strength is required. In footwear, for example, after the application of the adhesive and the creation of the bond between the sole and the upper, the next stage is that of removing the aspect last used to shape the footwear. This step occurs shortly after bonding and generates considerable stress on the adhesive bond, so a minimum adhesion of at least 1 N/mm after the joint formation is required [34]. Some factories use a cooling tunnel just after the adhesive bond is made to reduce the temperature of adhesive bonding and, therefore, relax the stresses of all the materials present. In any case, this sudden cooling may favour the initial adhesion of the adhesive by facilitating the crystallization stage. For this reason, in this study, the initial adhesion was evaluated after 5 min of curing at room temperature, as well as after 5 min of being at −10 °C, simulating the conditions of the adhesively bonded materials after the cold tunnel.

Initial adhesion tests (Table 5), after 5 min at room temperature (RT) or after cooling (COOL), of the joints carried out with the PUR showed the lowest T-peel strength, with cohesive failure of the adhesive (C) in both cases, indicating poor initial adhesion, consistent with the primarily amorphous structure observed in DSC. Adding the TPU improves the initial adhesion, but it is the application of accelerated cooling that substantially increases the green strength. The effect is more marked in PUR-10, with 0.5 ± 0.1 N/mm at room temperature and 5 ± 1 N/mm after cooling. PUR-15 (15 wt.% TPU) exhibits a slightly lower T-peel strength after cooling, likely due to excessive crystallinity that may shorten the set time and open time for sufficient adhesion to develop [24].

Additionally, the final adhesion of the footwear materials joined with the synthesized adhesives was evaluated. T-peel strength values were evaluated after 7 days of curing at 23 °C and 50% relative humidity, and the results are included in Table 6. The values show increased T-peel strength in all cases, with the PUR reaching 12 ± 3 N/mm with a 100% adhesive failure mode. They still exceed the minimum strength requirements set by EN 15307:2014 [34] for upper-to-sole bonding: a minimum of 5 N/mm (3.5 N/mm with substrate failure) for high-demand footwear (e.g., mountain footwear) and 4 N/mm (3 N/mm with substrate failure) for medium-demand footwear (e.g., children’s and general-purpose sports shoes). The reduction in the T-peel strength for PUR-10 and PUR-15, compared to the PUR, may be attributed to an increase in crystallinity, which hinders moisture penetration into the system, which can reduce cross-linking [3] and lower flexibility and damping properties (as was shown in the DMA) because localized stress at the edge of the joint evaluated under T-peel forces tends to generate micro-fractures or failures, and if the adhesive is well damped, it can dissipate some of this concentrated energy, which reduces the likelihood of failure propagation and improves separation resistance.

However, both TPU-containing adhesives maintained strong bonding performance and 100% adhesive failure, demonstrating consistent and durable adhesion suitable for footwear applications. This balance of high initial adhesion and sufficient final bond strength makes these adhesives effective for industrial use, where both immediate and long-term adhesion are essential.

This level of final adhesion, combined with the good results observed after cooling, makes PUR-10 particularly suitable for footwear applications, where both initial and long-term bonding performance are essential. Although PUR-15 also shows good performance, PUR-10 provides a more balanced combination of flexibility, adhesive strength, and green strength, making it an optimal choice for demanding applications.

## 4. Conclusions

Reactive polyurethane hot-melt adhesives were synthesized from a blend of bio-based polycarbonate polyols and polypropylene glycol with 4,4′-diphenylmethane diisocyanate as an isocyanate. Different amounts of a thermoplastic polyurethane were incorporated to improve the green strength. The incorporation of 10 wt.% of the thermoplastic polyurethane was found to improve the initial adhesion, thermal resistance, and low-temperature flexibility. The incorporation of higher amounts (15 wt.%) also produced good adhesion results but resulted in flexibility similar to that of polyester/polyether-based reactive polyurethane hot-melt adhesive. On the other hand, the effect of sudden cooling of the adhesive on the initial and final adhesion strength was evaluated, and it was found that it improves the green strength with some detriment to the final adhesion.

## Figures and Tables

**Table 1:** Composition (wt.%) of the PURs.

Raw Material	PUR	PUR-10	PUR-15
Polypropylene glycol	31.87	28.66	27.06
Bio-based polycarbonate diol	31.87	28.66	27.06
TPU	0.00	10.07	15.10
4,4′-MDI	36.26	32.61	30.78

**Table 2:** Main results obtained from the DSC thermograms of the HMPURs.

Adhesive	Tg (°C)	Tm (°C)	ΔHm (J/g)
TPU	−18.76	57.99	−44.65
PUR	−5.41	-	-
PUR-10	−18.02	50.91	−1.22
PUR-15	−15.45	49.91	−4.43

**Table 3:** DTG and TG data of adhesives after curing.

Adhesive	T1 (°C)	Wt1 (wt.%)	T2 (°C)	Wt2 (wt.%)	Residue (wt.%)
PUR	319	80.97	425	13.54	11.48
PUR-10	330	80.57	454	13.71	7.18
PUR-15	331	81.94	448	13.47	5.76

**Table 4:** DMA results. Storage modulus (E′) at 25 °C and −40 °C, the temperatures of the Tg and β transitions of the adhesives.

Adhesive	E′ (MPa) at 25 °C	E′ (MPa) at −40 °C	Tg (°C)	T_β_ (°C)
PUR	39	252	36	-
PUR-10	25	620	27	70
PUR-15	866	2689	39	59

**Table 5:** T-peel strength test results, initial adhesion.

Adhesive	5 Min RT		5 Min COOL	
	Peel Strength (N/mm)	Failure	Peel Strength (N/mm)	Failure
PUR	0.06 ± 0.03	100 C	0.6 ± 0.1	100 C
PUR-10	0.47 ± 0.06	100 C	5.23 ± 1.26	100 C
PUR-15	0.41 ± 0.06	100 C	1.83 ± 1.14	100 C

C: separation in the adhesive film without unsticking, defective cohesion [23].

**Table 6:** T-peel strength test results, final adhesion.

Adhesive	7 Days RT	
	Peel Strength (N/mm)	Failure
PUR	12.48 ± 2.60	100 A2
PUR-10	8.59 ± 1.52	100 A2
PUR-15	8.05 ± 1.07	100 A2

A2: separation of the adhesive film from the sole material, defective adhesion [23].

## Data Availability

The original contributions presented in the study are included in the article, further inquiries can be directed to the corresponding author.
